# Perpetrators and victims of cyberbullying among youth with conduct disorder

**DOI:** 10.1007/s00787-022-01973-0

**Published:** 2022-03-29

**Authors:** Sarah Baumann, Anka Bernhard, Anne Martinelli, Katharina Ackermann, Beate Herpertz-Dahlmann, Christine Freitag, Kerstin Konrad, Gregor Kohls

**Affiliations:** 1 Department of Child and Adolescent Psychiatry, Psychosomatics and Psychotherapy, University Hospital RWTH Aachen, Neuenhofer Weg 21-22, 52074 Aachen, Germany; 2 Department of Child and Adolescent Psychiatry, Psychosomatics and Psychotherapy, University Hospital Frankfurt, Frankfurt am Main, Germany; 3 Psychology School, University of Applied Science Fresenius, Idstein, Germany; 4 Department of Education, University of Hamburg, Hamburg, Germany; 5 Child Neuropsychology Section, Department of Child and Adolescent Psychiatry, Psychosomatics and Psychotherapy, University Hospital RWTH Aachen, Aachen, Germany; 6 JARA-Brain Institute II, Molecular Neuroscience and Neuroimaging, RWTH Aachen and Research Centre Jülich, Jülich, Germany; 7 Department of Child and Adolescent Psychiatry, Faculty of Medicine, TU Dresden, Dresden, Germany

**Keywords:** Conduct disorder, Aggression, Cyberbullying, Victimization, Perpetration

## Abstract

Due to modern technological innovations, aggressive behaviors have expanded into the cyberspace, creating a new matter of public concern: cyberbullying. Antisocial and aggressive behaviors, including bullying are characteristic for children and adolescents diagnosed with conduct disorder (CD), raising the question whether these youths are highly involved in cyberbullying experiences, too. 206 participants with CD versus typically developing controls (TDCs) aged 9–19 years (57% girls) were included in the study. Individuals completed several self-report measures investigating cyber- and traditional bullying experiences, and hierarchical multiple regression analyses were conducted to explain the relationship between cyberbullying victimization and perpetration with demographic and clinical variables. Experiences of cyberbullying victimization and perpetration were significantly higher among youth with CD compared to TDCs, and this was accompanied by significantly higher scores on a measure of traditional bullying in CD versus TDCs. CD diagnosis, female sex and higher levels of callous-unemotional (CU) traits were each uniquely associated with increased experiences of cyberbullying victimization, whereas CD diagnosis, higher levels of CU traits and older age were each uniquely associated with increased experiences of cyberbullying perpetration. Individuals with CD, compared to TDCs are at higher risk of becoming cyberbully victims and perpetrators, hence representing an important novel aspect in the assessment and treatment of these youths.

**Supplementary Information:**

The online version contains supplementary material available at 10.1007/s00787-022-01973-0.

## Introduction

Aggression and other types of antisocial behaviors are a leading cause for children and youth to be referred to mental health services, of school drop-out, illegal drug consumption, and—in females—teenage pregnancy, thereby generating enormous financial costs to society [1]. One of the most prevalent externalizing disorders in youth is conduct disorder (CD), which is characterized by a repetitive and persistent pattern of antisocial behaviors that violate the basic rights of others and major age-appropriate rules and societal norms [2]. Aggressive behaviors that are required for a diagnosis of CD range from overt or direct aggression, such as physical cruelty to people and/or animals, to more covert or indirect forms of aggression, including threatening, intimidating or bullying others. It has been suggested that bullying behaviors might be the result of social learning processes, such as modeling or imitating others [3] and hence individuals with CD might have internalized that aggressive strategies (including bullying) are useful in solving potential conflicts.

Bullying is a type of aggressive behaviors “intended to inflict injury or discomfort upon another individual” [4], postulating a relationship between two or more individuals characterized by an imbalance of power, with victims being exposed repeatedly and over time to the negative actions of one or more individuals [5]. To date, most relevant research has mainly focused on bullying behaviors observed in rather “traditional” social contexts (e.g., school). However, due to modern technological innovations and hence the possibility of having social contacts in virtual environments, aggressive behaviors have expanded into the cyberspace, creating another form of bullying behavior, known as “cyberbullying” [6].

Cyberbullying is defined as aggression that is intentionally and repeatedly carried out via electronic means, such as emails, chats or text messages and intended to harm others that cannot easily defend themselves [7, 8]. According to a review of Livingstone and Smith [9], prevalence estimates for cyberbullying vary substantially, depending on the demographic characteristics of the sample investigated (e.g., age, sex/gender, geographic region/country) and the definition and operationalization of cyberbullying used. Population-based studies globally report prevalences that range from 10 to 40% for cyberbullying victimization and from 3 to 50% for cyberbullying perpetration [6]. Interestingly, cyberbullying and traditional bullying experiences are highly associated with each other, such that 9 out of 10 adolescents who, for instance, report experiences of cybervictimization are also victims of traditional forms of bullying (e.g., Wolke et al. [10]).

In fact, cyberbullying behaviors appear to be very similar to traditional bullying behaviors, including direct/overt aggressive acts, such as stalking or threatening somebody physically, as well as indirect/relational aggression like compromising somebody’s integrity on social platforms [7, 8]. However, cyberbullying can be performed in absolute anonymity [6], making it easier to act aggressively through (fake) e-mail-accounts, text messages or online posts, because the cyberspace separates perpetrator and victim physically, whereby chances for remorse of the perpetrator or empathy towards their victim are significantly reduced [11]. Moreover, cyberbully perpetrators are rarely confronted with behavioral consequences [12], as many children and adolescents use their computers or smartphones without any regulatory supervision by parents or teachers [6, 13]. Finally, cyberbullying can be practiced at any times and everywhere, without being restricted to specific environments (e.g., the schoolyard) [6].

Although cyberbullying seldom has serious consequences for the perpetrator, it has a serious negative impact on the mental health of the victim, ranging from lowered self-esteem [10, 14] to school adjustment problems and development of depressive symptoms [7], and it even can lead to suicidal ideations and behaviors [8]. Consequently, it is necessary to identify those youths who are inclined to engage in (cyber) bullying behaviors to protect children and adolescents who are their potential victims.

Research on traditional bullying has consistently shown that boys engage in bullying behaviors more often and overtly than girls [6]. There are, however, indications—notably with some mixed findings—that girls are more likely than boys to experience cyberbullying [15]. In a population-based study on cyberbullying experiences among adolescents aged 13–16 years, it was shown that girls were more likely than boys to be cyberbully victims (6% vs. 3.5%), but were less likely than boys to be cyberbully perpetrators (5.6% vs. 9.3%) or cyberbully victim/perpetrators (4.6% vs. 5.8%) [11]. However, other studies report fewer gender differences, particularly with regard to cybervictimization [16]. The finding that girls may be more often the victims of cyberbullying compared to boys could result from a greater usage of online social networks among female youth, providing them not only with more opportunities than male youth to become involved in cyberbullying victimization and/or perpetration but also making them more susceptible to the damaging consequences of cyberbullying victimization [17].

As boys and girls with CD are characterized by antisocial and aggressive behaviors, including bullying [2], it can be expected that these youths are similarly involved in experiences with cyberbullying perpetration and potentially cyberbullying victimization, too [18]. In a study by Coolidge et al. [19] it was shown that greater externalizing psychopathology [i.e., oppositional defiant disorder (ODD), attention-deficit/hyperactivity disorder (ADHD) and CD] was related to more bullying behaviors among youth, and this in turn was correlated with executive function deficits (i.e., decision-making, planning, learning, and social judgment)—processes that depend largely on the appropriate functioning of frontal brain regions. In an attempt to build a theoretical framework on the emergence of bullying behaviors it has been proposed that frontal brain regions are crucial for the development of social behaviors including the inhibition of inappropriate behavior, such as aggressiveness [20]. Similarly, the social emotional development (SED) model [21] illustrates that social behaviors depend on a person’s ability to understand, express, and regulate their emotions to develop and maintain ‘healthy’ social relationships, whereas deficient SED has been shown to be associated with greater bullying involvement, including victimization and perpetration [22, 23]. Indeed, in a recent large-scale study on emotion functioning by Kohls et al. [24], it was found that, compared to typical controls, children and adolescents with CD were impaired in emotion recognition, learning and regulation, possibly contributing to the emergence and maintenance of antisocial behaviors. Moreover, feelings of empathy and remorse are significantly reduced in children and adolescents who have callous-unemotional (CU) traits (i.e., a lack of guilt, remorse and empathy) which often accompanies CD [2], making those youths even more susceptible for (cyber) bullying experiences.

However, to our knowledge, to date there are no scientific studies available that have investigated the relationship between CD and cyberbullying, and hence there are no data to confirm that youths with CD are indeed more often involved in cyberbullying experiences than typically developing children (TDCs). In a study by Fanti et al. [25], potential risk and protective factors of cyberbullying and cyberbullying victimization were investigated longitudinally. It was shown that higher levels of CU traits were associated with cyberbullying behaviors. Moreover, Fanti and Kimonis [26] explored traditional bullying in a community sample of youths with conduct problems and CU traits, revealing that bullying was highest among youths who scored high on narcissism, impulsivity, or conduct problems, while victimization by peers was associated with high levels of impulsivity. Similarly, Viding et al. [27] investigated the relationship between CU traits and conduct problems in a community sample of youths to identify potential risk factors of engaging in traditional bullying. Results showed that (i) higher levels of CU traits were associated with higher levels of direct bullying, (ii) a combination of CU traits and conduct problems appeared to be a risk factor for both direct (e.g., threatening another person) and indirect bullying (e.g., social exclusion), and (iii) boys engaged more often in direct bullying, whereas girls exhibited more often indirect bullying.

However, all of the above-mentioned studies included groups of children and adolescents with conduct problems and/or varying levels of CU traits, clearly limiting the generalizability to children and adolescents with a full-blown CD diagnosis. Moreover, only one study [25] investigated cyberbullying, whereas the remaining studies explored bullying behaviors in traditional social settings, which may not be adequately comparable to cyberbullying. To address these research gaps, the primary aim of the current study was to investigate whether children and adolescents with CD are more frequently involved in cyberbullying experiences than TDCs. We analyzed data collected through several self-report measures from clinically well-assessed girls and boys with CD and TDCs. As our sample is a subsample derived from the FemNAT-CD database [28], results of the larger sample included, among others, sex differences in comorbidity patterns and clinical presentations, including CU traits and post-traumatic stress disorder [29], as well as in relational aggression [30].

We expected to find that (1) the proportion of cyberbully perpetrators as well as victims would be significantly higher in youths with CD than TDCs, (2) higher levels of CU traits, irrespective of group status, would be a predictor of cyberbullying perpetration, and (3) female sex, irrespective of group status (CD or TDCs), would be a predictor of cyberbullying victimization. We additionally expected to find associations between experiences with cyberbullying and experiences with bullying in “traditional” contexts (i.e., at school).

## Methods

### Participants

206 participants, aged 9–19 years, were recruited in Frankfurt and Aachen (Germany) through community outreach, mental health clinics, welfare institutions and youth offending services (e.g., by presentations at schools and institutions, distributing flyers, or telephone contact of former study participants who agreed to be recontacted) to participate in this cross-sectional study (CD: *n* = 76 and TDCs: *n* = 130) (Table 1). This sample is a subsample derived from the FemNAT-CD study [28]. Overall exclusion criteria of the study were autism spectrum disorder, psychosis or schizophrenia, mania or bipolar disorder, genetic syndromes, neurological disorders and an IQ < 70 to ensure that symptoms of severe psychiatric disorders (e.g., hallucinations, delusions or thought disorders) or genetic syndromes, neurological dysfunctions and intellectual disability would not interfere with the participants’ ability to understand and accomplish the assessments. Participants of the clinical group fulfilled current criteria for CD, whereas participants of the TDC group were free of any current psychiatric diagnoses and had no life-time diagnoses of CD, ODD or ADHD. All diagnoses were based on DSM-IV-TR criteria [31]. Participants who were taking psychotropic medication were tested while on medication. The study protocol was approved by the local ethics committees. Participants and their caregivers gave written informed consent and were compensated monetarily for their participation.

**Table 1:** Sample demographics and clinical characteristics

	CD *N* = 76	TDC *N* = 130	Group (CD vs. TDC) t/*X*^2^#	Cohen’s *d*
Sex (f/m)	44/32	74/56	0.0	
Age (years)	14.0 (2.0)	14.8 (1.8)	3.0**	− 0.43
Estimated IQ	97.9 (10.0)	102.5 (13.1)	2.8*	− 0.38
CB-V	4.2 (7.6)	1.2 (2.9)	3.3**	0.58
CB-P	1.0 (1.8)	0.3 (1.2)	3.0**	0.48
SAHA-bully (total sum)	15.7 (7.5)	11.6 (3.1)	4.3***	0.79
ICU (total sum)	27.1 (10.6)	18.9 (7.7)	5.8***	0.92
Psychotropic medication *n* (%)			62.4***	
Neuroleptics	1 (1.3)	N/A		
Stimulants	22 (28.9)	N/A		
Antidepressants	8 (10.5)	N/A		
Comorbid diagnoses *n* (%)				
ODD	66 (86.8)	N/A		
ADHD	43 (56.6)	N/A		
MDD	16 (21.1)	N/A		
Anxiety disorders	14 (18.4)	N/A		
PTSD	9 (11.8)	N/A		
SUD	11 (14.5)	N/A		

*ADHD* attention-deficit/ hyperactivity disorder,* CB-V* cyberbullying victimization,* CB-P* cyberbullying perpetration,* CD* conduct disorder,* f/m* female/male,* ICU* inventory of callous-unemotional traits,* IQ* intelligence quotient,* MDD* major depressive disorder,* N/A* not applicable,* ODD* oppositional defiant disorder,* PTSD* post-traumatic stress disorder,* SAHA-bully* social and health assessment-bully,* SUD* substance use disorder (including substance abuse and dependence*)*,* TDC* typically developing controls

Diagnoses are based on the Kiddie Schedule for Affective Disorders and Schizophrenia for School-Age-Children Present and Lifetime version (K-SADS-PL); CD (*n* = 5): no comorbid disorder

^*#*^*p* values are based on two-sample *t* tests (or *X*^2^ tests). *p ≤ 0.05; **p ≤ 0.01; ***p ≤ 0.001

### Assessments

All participants were clinically evaluated with the Kiddie Schedule for Affective Disorders and Schizophrenia for School-Age Children—Present and Lifetime Version [32]. The K-SADS-PL interview was administered by trained staff separately to participants and their caregivers, and clinical summary ratings were achieved to determine group allocation and to identify possible comorbid psychiatric diagnoses. As data were collected over several years, there were up to six independent, clinically trained staff (i.e., three per site) who evaluated the study participants. Inter-rater reliability (IRR; *N* = 75) was high for CD current episode (Cohen’s kappa = 0.91, 95% agreement, *n* = 75). IRR of other mental disorders, including ADHD, ODD, major depressive disorder (MDD), and generalized anxiety disorder (GAD), was also high (Cohen’s κs ≥ 0.84, agreement rates ≥ 92%). Concurrent validity of the diagnoses assessed with the K-SADS-PL has been reported by Kaufmann et al. [32].

Full-scale IQs were estimated using the vocabulary and matrix reasoning subtests of the Wechsler Intelligence Scale for Children-Fourth Edition [33], or the Wechsler Intelligence Scale for Adults-Fourth Edition [34]. The vocabulary subtest consists of 31 items and individuals are asked to define a word or concept that is verbally presented to them. Answers are scored according to a manual on a 0–2 point basis. The matrix reasoning subtest includes 30 different, visually presented, incomplete matrices and individuals are asked to complete the matrix by choosing one of five visually presented options that correctly completes the matrix. Each correct answer is rated with 1 point. The internal consistency and test–retest reliability were reported to be excellent (*α* > 0.90) [35].

To assess participants experiences with cyberbullying victimization and perpetration we used a self-report measure called “Erfahrungen mit Cybermobbing” (ECM; English: Experiences with Cyberbullying), which was adapted from a community screening instrument developed by Sitzer et al. [12]. After a short description of the term “cyberbullying”, participants are asked to share their experiences with cyberbullying victimization (CB-V) and perpetration (CB-P). It was explicitly stressed that there are not any right or wrong answers, and participants were instructed to answer each question distinctly. The ECM is divided into six different parts, referring to (1) experiences with CB-V, (2) coping strategies in response to CB-V, (3) possible reasons for CB-V, (4) experiences with CB-P, (5) reasons for CB-P, and (6) consequences of CB-P. In parts 1 and 4 (i.e., experiences with CB-V and experiences with CB-P), 15 different questions were used to assess how often participants were exposed to cyberbullying themselves or how often they cyberbullied another person via the internet or mobile phone during the past three months (e.g., CB-V: *“How often have you been insulted, mocked or threatened?”*; CB-P: *“How often did you insult, mock or threaten another person?”*) and possible answers are given on a five-point Likert scale ranging from “never” (0) to “several times per week” (4). Both scales showed good internal consistency (Cronbach’s *α* = 0.90 and 0.71, respectively), and sum scores were created for both subscales, separately. Based on the work by Sitzer et al. [12], we classified our participants into four different groups: (1) CB-victim: a score of 1 or higher on at least one of the 15 CB-V items, (2) CB-perpetrator: a score of 1 or higher on at least one of the 15 CB-P items, (3) CB-victim-perpetrator: a score of 1 or higher on at least one of the 15 CB-V items and one of the 15 CB-P items and (4): CB-neutral: a score of 0 on all 30 items.

The Social and Health Assessment—bullying questionnaire (SAHA—bully) [36] is a nine item self-report measure that assesses experiences with bullying victimization in school during the last school year (e.g., *“Someone tried to get me into trouble with my friends”*), rated on a four-point Likert scale ranging from “Not at all” (1) to “4 or more times” (4). Summary scores were created using all nine items, and internal consistency was good (Cronbach’s *α* = 0.88).

We used the self-report measure of the Inventory of Callous-Unemotional traits (ICU) [37] which is a 24-item questionnaire, rated on a four-point Likert scale ranging from “not at all true” (0) to “definitely true” (3), where higher scores represent higher levels of psychopathy. We used the total sum score of the subdimensions (1) uncaring, (2) callousness and (3) unemotionality [38] (Cronbach’s *α* = 0.77).

### Statistical analyses

We compared groups on demographic and clinical variables using chi-squared and two-sample *t* tests (SPSS v26.0; IBM Corp., Armonk, NY). Pearson’s correlations were used to identify relationships between cyberbullying victimization (CB-V) as well as perpetration (CB-P) and demographic variables (i.e., group, sex, age, and IQ) as well as sum scores for clinical measures (i.e., ICU, SAHA-bully). Two separate hierarchical multiple regression analyses were conducted to construct models explaining the relationship between the outcome variables (1) CB-V and (2) CB-P and the investigated factors. For both outcome variables, group (0 = TDC, 1 = CD), sex (0 = male, 1 = female), age and ICU total sum score were entered in step 1 and the interaction term for group and sex (group × sex) was entered in step 2. For all analyses, the alpha level was set at 0.05. Effect sizes were calculated using Cohen’s *d* for *t* tests where 0.2, 0.5, and 0.8 represent small, medium and large effects, respectively [39].

## Results

### Demographic and clinical characteristics, including (cyber)bullying experiences

**Fig. 1:**
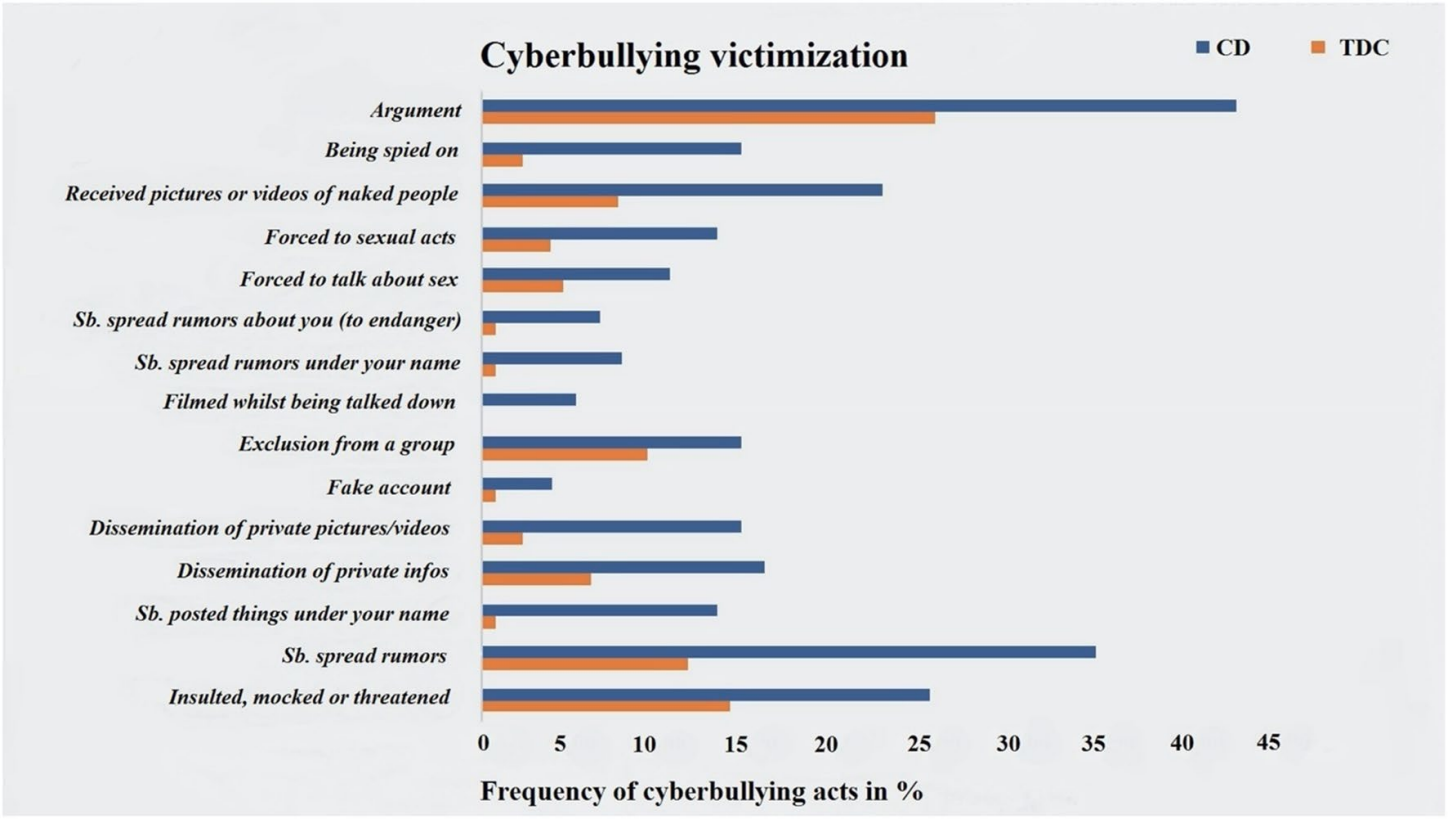
Experiences of youths identified as cyberbully victims. Only participants, who scored ≥ 1 on at least 1/15 ECM victimization items were included

**Fig. 2:**
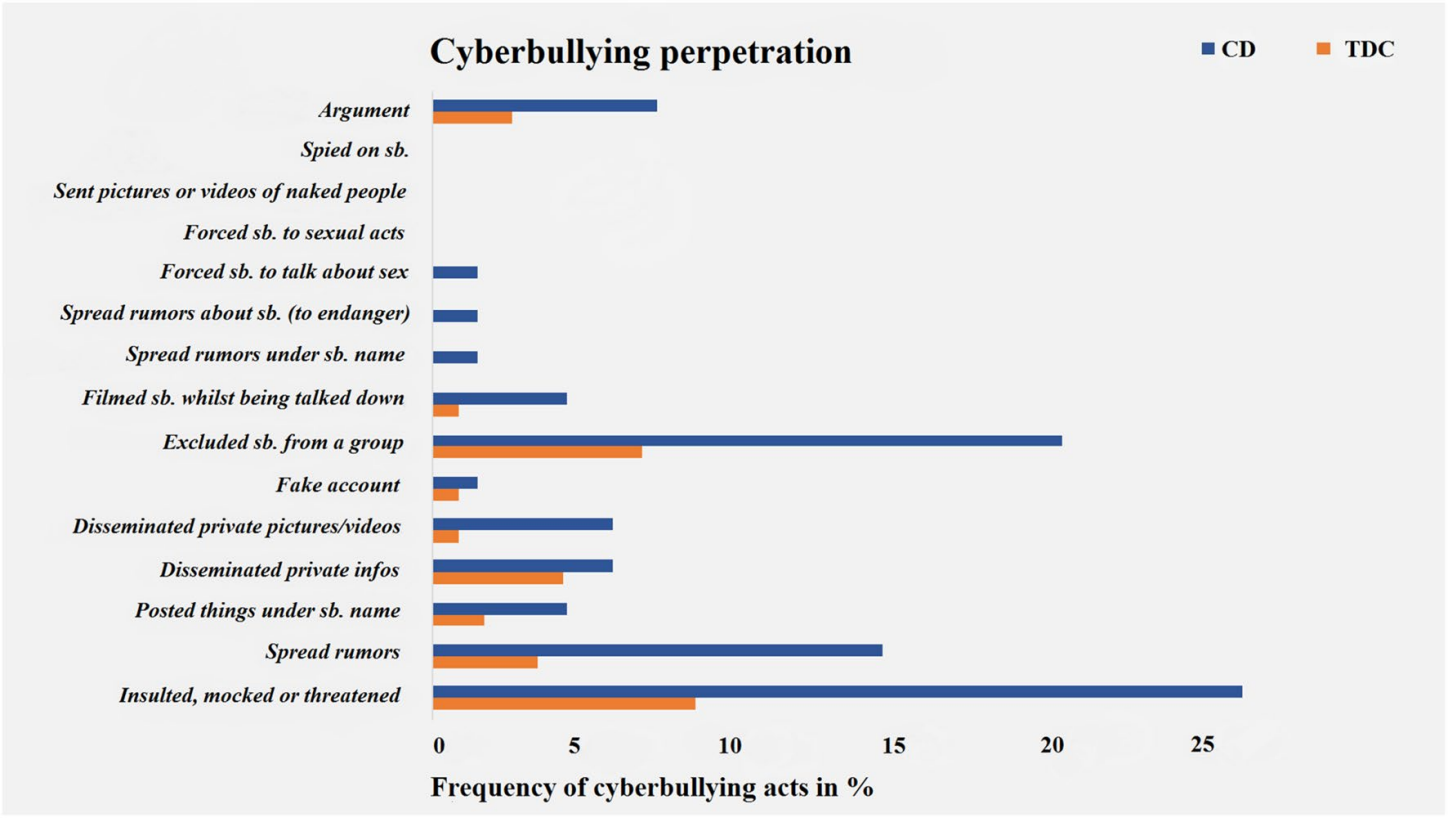
Experiences of youths identified as cyberbully perpetrators. Only participants, who scored ≥ 1 on at least 1/15 ECM perpetration items were included

Sexes were equally distributed across groups, although youths with CD were significantly younger and had significantly lower IQs than TDCs. As expected CD youths showed significantly higher levels of CU traits compared to TDCs. Experiences of cyberbullying victimization and perpetration were both significantly higher among individuals with CD compared to TDCs and accompanied by significantly higher experiences of traditional bullying (i.e., SAHA-bully) in CD versus TDCs. Descriptively, experiences of cyberbullying victimization most frequently included: (i) getting into an argument, (ii) being exposed to rumor spreading, and (iii) being insulted, mocked or threatened (see Fig. 1). By contrast, experiences of cyberbullying perpetration most commonly involved: (i) insulting, mocking or threatening somebody, (ii) excluding somebody socially, and (iii) spreading rumors about somebody (see Fig. 2).

### Correlational analyses

Cyberbullying victimization (CB-V) was weakly positively correlated with group (*r* = 0.27), sex (*r* = 0.16), and CU traits (*r* = 0.25), weakly negatively correlated with IQ (*r* = 0.17), and moderately positively correlated with experiences of traditional bullying victimization (*r* = 0.44). Cyberbullying perpetration (CB-P) was weakly positively correlated with group (*r* = 0.23) and experiences of traditional bullying victimization (*r* = 0.21) and moderately positively correlated with CU traits (*r* = 0.30) (Table 2).

**Table 2:** Zero-order Pearson correlations of main variables

Variable	1	2	3	4	5	6	7	8
Dependent variables								
1. CB-V	–	0.33**	0.27**	0.16*	0.06	− 0.17*	0.25**	0.44**
2. CB-P	0.33**	–	0.23**	0.06	0.10	− 0.12	0.30**	0.21**
Demographic variables								
3. Group	0.27**	0.23**	–	0.01	0.40	0.14	0.10	0.00
4. Sex	0.16*	0.06	0.01	–	0.14*	− 0.18*	− 0.13	− 0.02
5. Age	0.06	0.10	− 0.20*	0.14*	–	− 0.26**	− 0.03	− 0.26**
6. IQ	− 0.17*	− 0.12	− 0.18**	− 0.18*	− 0.23**	–	− 0.15*	0.08
Clinical measures								
7. ICU	0.25**	0.30**	0.41**	− 0.13	− 0.03	− 0.15*	–	0.23**
8. SAHA-bully	0.44**	0.21**	0.36**	− 0.02	− 0.26**	0.08	0.23**	–

*CB-V* cyberbullying victimization,* CB-P* cyberbullying perpetration,* ICU* inventory of callous-unemotional traits,* IQ* intelligence quotient,* SAHA-bully* social and health assessment-bully

*p ≤ 0.05, **p ≤ 0.01

### Multiple regression analyses

The model for cyberbullying victimization (CB-V) was significant [*F*(4,196) = 8.24, *p* < 0.001], with an *R*^2^ of 0.14 (Table 3). The scores on cyberbullying victimization of youths with CD were 2.63 points higher than for TDCs (*p* < 0.002), controlling for sex, age, and CU traits. Sex (*p* < 0.03) and CU traits (*p* < 0.03) were each independent predictors with a respective increase on victimization scores of 1.63 points for females and 0.09 points per point increase in CU score for youths with higher CU traits when holding the other factors constant. There was no significant influence of age (*p* = 0.21). As the interaction term of group by sex did not cause a significant change in *F*(*p* = 0.14), the interaction term was excluded from the model.

**Table 3:** Multiple regression analyses of cyberbullying victimization and perpetration

Variable	*B*	*SE B*	*ß*	*t*	*p*
Victimization					
(Constant)	− 4.94	2.72		− 1.82	ns
Group	2.63	0.79	0.25	3.32	**0.001**
Sex	1.63	0.69	0.16	2.35	**0.02**
Age	0.23	0.18	0.09	1.25	ns
ICU	0.09	0.04	0.17	2.35	**0.02**
Perpetration					
(Constant)	− 2.10	0.81		− 2.60	0.01
Group	0.50	0.23	0.16	2.10	**0.04**
Sex	0.23	0.21	0.08	1.11	ns
Age	0.11	0.05	0.14	1.98	**0.05**
ICU	0.04	0.01	0.25	3.38	**0.001**

Bold values indicate significant results of main variables of interest

*ß* standardized beta,* B* unstandardized beta,* ICU* inventory of callous-unemotional traits,* SE B* standard error of unstandardized beta,* t* *t* test statistic

The model for cyberbullying perpetration (CB-P) was also significant [*F*(4,196) = 7.32, *p* < 0.001], with an *R*^2^ of 0.13. The scores on cyberbullying perpetration of youths with CD were 0.50 points higher than for TDCs (*p* = 0.04), controlling for sex, age, and CU traits. CU traits (*p* = 0.001) and age (*p* = 0.05) were each independent predictors with a respective increase on perpetration scores of 0.04 per point increase in CU score for youths with higher CU traits and 0.11 points per year increase in age for individuals who were older when holding the other factors constant. There was no significant influence of sex (*p* = 0.27). Again, the interaction term of group by sex did not cause a significant change in *F*(*p* = 0.61), so the interaction term was excluded from the model.

## Discussion

The primary aim of this study was to investigate whether children and adolescents with a clinical diagnosis of CD are more often involved in cyberbullying experiences than TDCs. Our results showed that both experiences of cyberbullying victimization and perpetration were significantly higher among youths with CD compared to TDCs, and this was accompanied by significantly higher scores on a measure of traditional bullying in CD youths versus TDCs. Moreover, our analyses revealed that CD diagnosis, female sex and higher levels of CU traits were each uniquely associated with increased experiences of cyberbullying victimization, whereas CD diagnosis, higher levels of CU traits and older age were each uniquely associated with increased experiences of cyberbullying perpetration. Rates of cybervictimization and -bullying were generally high also in TDC with ~ 38% of all TDC youth reporting to have cyberbullying experiences (see Table S2). This rate, however, is in line with population-based studies globally which report prevalences that range from 10 to 40% for cybervictimization and from 3 to 50% for cyberperpetration [6].

In line with our first hypothesis, proportions of experiences with cyberbullying victimization and perpetration were significantly higher among youths with CD than TDCs. The fact that CD youths perpetrate others in the cyberspace significantly more often than TDCs might not be surprisingly, as these youths are characterized by antisocial and aggressive acts [2]. In the large-scale study on emotion functioning by Kohls et al. [24], it was found that, compared to typical controls, children and adolescents with CD were impaired in emotion recognition, learning and regulation, possibly contributing to the emergence and maintenance of antisocial behaviors. Adequate emotion functioning skills, such as identifying and processing other people’s emotional expressions, are pivotal for daily interpersonal communication [40]. The anonymity of the cyberspace and the lack of emotional capacity among CD youth may make them much less likely to feel empathy or remorse [11]. Additionally, our CD sample showed high levels of CU traits (see Table 1), leaving them with a lack of concern for other peoples’ feelings [41], possibly enhancing and maintaining antisocial and aggressive acts online, such as cyberbullying. The significantly higher proportion of experiences with cyberbullying perpetration among the CD group relative to TDCs might also be associated with our finding of a significantly higher proportion of experienced cyberbullying victimization among CD youths compared to TDCs. In a recent study of Liu et al. [18] cyberbullying victimization and perpetration were investigated in a sample of adolescents with a clinical diagnosis of ADHD, a highly comorbid condition of CD [42]. They found that frustration intolerance increased the risks of becoming cyberbully perpetrators and victims. The authors concluded that youths with ADHD and high levels of frustration intolerance compensated their frustration by perpetrating cyberbullying and that those cyberbullying acts in turn might have provoked others to fight back and hence increased the risk for the perpetrators to become cyberbully victims themselves. A substantial number of CD youths in our sample reported being bullied in school or via the internet because of their own cyberbullying perpetrating behavior (see Fig. S1). This is in line with the conclusions of the study by Liu et al. [18] and hence supporting our results of significantly higher rates of experiences with cyberbullying victimization among CD youths relative to TDCs.

In line with our second hypothesis and the results reported by Fanti et al. [25], higher levels of CU traits were a significant predictor of cyberbullying perpetration. Individuals with high levels of CU traits appear to not notice the fear and distress experienced and expressed by their victims [43] and consequently would not inhibit their aggressive behavior. Moreover, youths with high levels of CU traits have been shown to expect that their aggressive behavior will result in a positive outcome, for instance ensuring one’s dominant position in a peer group [44], which may reinforce their aggressive behavior.

We also found that higher levels of CU traits predicted cyberbullying victimization. This could be because a substantial number of individuals in our sample were cyberbully victims and perpetrators concurrently (see Table S2). Research has shown that individuals who are “traditional” bully-victims have higher CU traits than sole victims of bullying or neutrals [27, 45]. Additionally, we found that older age was a significant predictor of becoming a cyberbully perpetrator. This might be the result of older children having more access to social media opportunities than younger ones, and they are possibly under less supervision of their parents and/or teachers.

Finally, our results showed that female sex was a significant predictor of cyberbullying victimization, which is consistent with previous research [10, 46, 47]. One reason for girls being more often identified as cyberbully victims could be that cyberbullying itself is of more indirect nature (i.e., covert and not face-to-face) and hence closely linked to relational aggression (i.e., socially excluding others, or spreading rumours). Relational aggression is a subtype of aggressive behavior that has been more frequently observed in females than males [46], including girls with CD [30]. Moreover, it has been proposed that girls are more often cyberbully victims than boys because of a greater usage of online social networks among female youth, providing them not only with more opportunities than male youth to become involved in cyberbullying victimization but also making them more susceptible to the damaging consequences of negative cyberbullying experiences [17]. Unfortunately, we did not ask our participants whether they knew their cyberbully perpetrator(s) or whether that person was of female or male sex. Smith et al. [46] previously reported that in their group of cyberbully victims, girls were bullied by girls as or more often than by boys, but there were no significant gender differences with regard to cyberperpetration, which partly confirms our finding that sex was not a significant predictor of cyberbullying perpetration. The lack of gender differences in cyberbullying perpetration may suggest a greater involvement of girls in this type of bullying behavior in contrast to traditional bullying contexts, where boys appear to predominate [6]. However, there exist mixed findings with regard to gender differences in cyberbullying perpetration, either reporting higher rates in boys than girls [47, 48] or no gender effects [49, 50], but no study has yet reported higher perpetration rates in girls compared to boys. The mixed findings may be due to different moderating effects, such as the expression of specific traits (i.e., CU traits) or even developmental aspects related to age, which was indeed a significant predictor for cyberbullying perpetration in the current sample. With respect to emotion processing skills, Kohls et al. [24] did not find any significant differences between boys and girls with CD, which might explain why we did not find any gender differences regarding cyberbullying perpetration in our sample. This idea could be explored in follow-up studies, especially since previous work has shown that deficits in social emotional competence (SED model; [21]) appear to be associated with traditional bullying behaviors depending on gender (e.g., [22, 23]).

The study had several strengths: our participants were extensively clinically assessed and reliably diagnosed, generating a relatively large sample of children and adolescents who fulfilled diagnostic criteria for CD. Moreover, we were able to include a substantial number of girls with CD which is important in being able to generalize the results to the whole CD population, allowing to analyse for possible sex differences. Additionally, we used reliable and valid measures of traditional and cyberbullying experiences in addition to other established clinical measures.

However, our study had also some limitations: we exclusively included self-report questionnaires on experiences with (cyber) bullying, making our results vulnerable for socially desirable responses. However, as (cyber) bullying behaviors often appear without any awareness of parents or teachers, this format appears to be one which would yield close to realistic information on the given issue, although it would have been also possible and informative to ask classmates. Moreover, we employed a cross-sectional study design, including a relatively narrow time window of three months for cyberbullying experiences to take place. Thus, our results are not generalizable to life-time (cyber) bullying experiences in children and adolescents and thus might actually underrepresent the number of affected individuals. For a measure of traditional bullying experiences, we used the SAHA-bully questionnaire which only considers experiences with victimization but not perpetration. Hence, our results with respect to traditional bullying experiences are limited by the perspective of victims and not generalizable to the experiences of perpetrators. Lastly, our cut-off used for identifying youths as cyberbully victims and/or perpetrators was relatively low, but previous studies have used comparable cut-offs [12] and have demonstrated its usefulness in categorizing the investigated individuals. Furthermore, the vast majority of our results are based on dimensional measures and the specified cut-offs were rather used for descriptive purposes.

In conclusion, the current findings provide evidence that youths with CD are significantly more often both victims and perpetrators of cyberbullying as well as victims of traditional bullying, compared to TDCs. Moreover, a CD diagnosis, female sex and higher level of CU traits increase the likelihood of becoming a cyberbully victim, whereas CD diagnosis, higher level of CU traits and age increase the likelihood of becoming a cyberbully perpetrator. Clearly, these findings have important implications for bullying prevention and intervention programmes, as to date, most programmes specifically target bullying in “traditional” contexts, such as schools. However, as our results clearly show, cyberbullying is a phenomenon that should not be underestimated, and since it frequently co-occurs with traditional bullying, prevention and intervention programmes need to address both contexts [10]. As CD youths were identified significantly more often as both cyberbully perpetrators and victims, clinicians need to pay careful attention to each of these issues when assessing and treating these youths. In addition, future studies are warranted to investigate the relationship between CD youths becoming cyberbully perpetrators and their experiences as cyberbully victims. This knowledge will allow developing prevention and intervention programmes that are specifically tailored to the circumstances and needs of the individual. Since research has shown that (cyber) victimization has a huge impact on adolescent mental health and social functioning [7], the field needs to focus on these issues to reduce potentially negative health outcomes among affected individuals in this digital age.

## Supplementary Information

Below is the link to the electronic supplementary material.

Supplementary file1 (DOCX 536 KB)
