# Supplementary material for: Perpetrators and victims of cyberbullying among youth with conduct disorder
Source: Eur Child Adolesc Psychiatry. 2022 Mar 29;32(9):1643–53. doi: 10.1007/s00787-022-01973-0 (PMC10460306; doi:10.1007/s00787-022-01973-0)

**Supplemental material for “Perpetrators and Victims of Cyberbullying among Youth with Conduct Disorder”**

Sarah Baumann^1^ (0000-0003-1854-0032), Anka Bernhard^2^, Anne Martinelli^3^ (0000-0002-7158-9778), Katharina Ackermann^4^, Beate Herpertz-Dahlmann^1^ (0000-0001-8450-3323), Christine Freitag^2^ (0000-0001-9676-4782), Kerstin Konrad^5,6^ (0000-0001-9039-2615), Gregor Kohls^5,7^ (0000-0003-2408-2939)

*^1^Department of Child and Adolescent Psychiatry, Psychosomatics and Psychotherapy, University Hospital RWTH Aachen, Aachen, Germany.*

*^2^Department of Child and Adolescent Psychiatry, Psychosomatics and Psychotherapy, University Hospital Frankfurt, Frankfurt am Main, Germany.*

*^3^ Psychology School, University of Applied Science Fresenius, Idstein, Germany.*

*^4^Department of Education, University of Hamburg, Hamburg, Germany.*

*^5^Child Neuropsychology Section, Department of Child and Adolescent Psychiatry, Psychosomatics and Psychotherapy, University Hospital RWTH Aachen, Aachen, Germany.*

*^6^JARA-Brain Institute II, Molecular Neuroscience and Neuroimaging, RWTH Aachen and Research Centre Juelich, Juelich, Germany*

*^7^Department of Child and Adolescent Psychiatry, Faculty of Medicine, TU Dresden, Dresden, Germany.*

**Corresponding author:** Sarah Baumann, MSc, Department of Child and Adolescent Psychiatry, Psychosomatics and Psychotherapy, University Hospital RWTH Aachen, Neuenhofer Weg 21-22, D-52074 Aachen, Germany. **Email:** sbaumann@ukaachen.de

**Table S1 Coping strategies of cyberbully victims.** Only participants, who scored ≥ 1 on at least 1/15 ECM victimization items were included

|  | **CD** | **TDC** | **Group** |  |  |  |
| --- | --- | --- | --- | --- | --- | --- |
|  | N= 41 | N= 48 | CD vs. TDC | |  |  |
|  | Median [95% CI] | Median [95% CI] | #U | Z | *p* | *r* |
| *Defensive strategies* |  |  |  |  |  |  |
| Suppressed thoughts | 2 [0, 2] | 1 [0, 2] | 915.0 | -.60 | .55 | -.07 |
| Ignored it | 3 [3, 4] | 3 [0.5, 4] | 861.0 | -1.10 | .28 | -.11 |
| Habituated | 2 [0.5, 3] | 1 [0, 2] | 920.5 | -.55 | .58 | -.05 |
| Waited | 2 [0, 3] | 0 [0, 1] | 809.5 | -1.60 | .12 | -.17 |
| Pretended everything would be fine | 2 [1, 3] | 0.5 [0, 2] | 774.5 | -1.81 | .07 | -.19 |
| Encouraged myself | 3 [0, 3] | 0 [0, 2] | 824.0 | -1.41 | .16 | -.15 |
| Made me sick | 0 [0, 1] | 0 [0, 0] | 674.5 | -3.63 | **.00** | **-.38** |
| *Proactive strategies* |  |  |  |  |  |  |
| Took vengeance | 0 [0, 0] | 0 [0, 0] | 924.5 | -.65 | .51 | -.07 |
| Tried harder being popular | 0 [0, 0] | 0 [0, 0] | 839.5 | -1.60 | .11 | -.17 |
| Technical provisions to protect | 0 [0, 0] | 0 [0, 0] | 909.5 | -.88 | .38 | -.09 |
| Searched help | 0 [0, 1] | 0 [0, 1] | 953.5 | -.29 | .78 | -.03 |
| Changed social environment | 0 [0, 0] | 0 [0, 0] | 820.5 | -2.10 | **.03** | **-.22** |
| *Accomodative strategies* |  |  |  |  |  |  |
| Others are worse off | 1 [0, 2] | 0 [0, 0] | 756.0 | -2.10 | **.04** | **-.22** |
| Became stronger | 2 [0, 2] | 1.5 [0, 2] | 974.5 | -.08 | .93 | -.09 |
| Can't be popular to everyone | 2 [0, 2.5] | 0.5 [0, 3] | 898.0 | -.75 | .45 | -.08 |

Note*: CD* conduct disorder*; CI* confidence interval*; r* pearson correlation coefficient*; TDC* typically developing controls*; U* Mann-Whitney-U-Test*; Z* standard score

**Table S2 Classification of ECM group status.** (1) CB-victim: a score of 1 or higher on at least one of the 15 CB-V items, (2) CB-perpetrator: a score of 1 or higher on at least one of the 15 CB-P items, (3) CB-victim-perpetrator: a score of 1 or higher on at least one of the 15 CB-V items and one of the 15 CB-P items and (4): CB-neutral: a score of 0 on all 30 items

|  | **CD** | **TDC** | **Group** |
| --- | --- | --- | --- |
|  |  |  | (CD vs. TDC) |
|  | N = 76 | N = 130 | *X^2^*# |
| *ECM group status n (%)* | |  | 15.4** |
| CB-victim | 17 (22.4) | 32 (24.6) |  |
| CB-perpetrator | 4 (5.3) | 2 (1.5) |  |
| CB-victim-perpetrator | 24 (31.6) | 16 (12.3) |  |
| CB-neutral | 31 (40.8) | 80 (61.5) |  |

Not*e: CB cyberbullying; CD* conduct disorder*; ECM* Erfahrungen mit Cybermobbing*; TDC* typically developing controls*; ***p≤ .01

**Figure S1** **Consequences of cyberbullying for youths identified as cyberbully perpetrators.** Only participants, who scored ≥ 1 on at least 1/15 ECM perpetration items were included


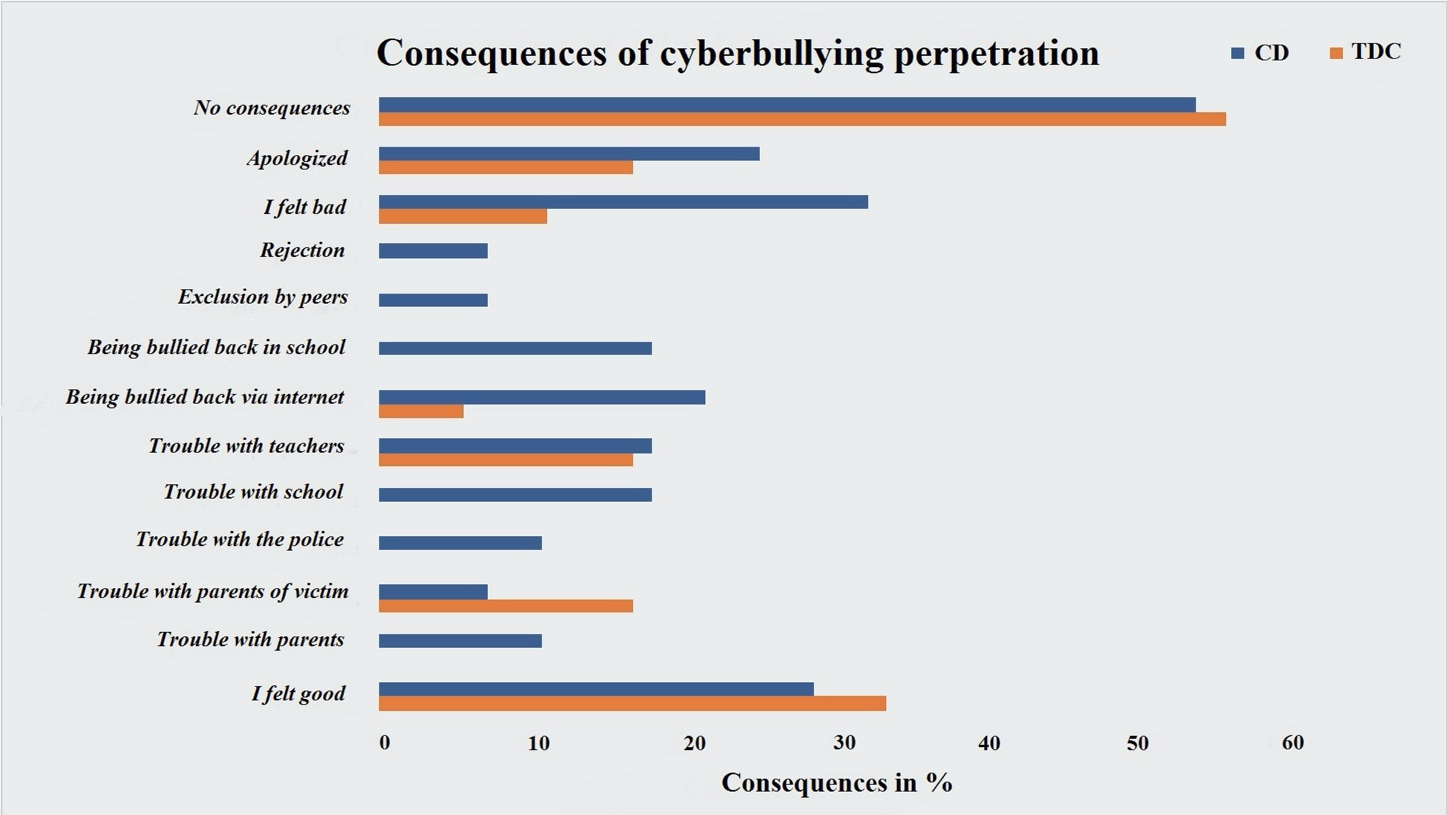

Supplement: Supplementary file 1 — Supplementary file1 (DOCX 536 KB) [file 787_2022_1973_MOESM1_ESM.docx]
